# A Model to Transform Communities Toward Becoming Dementia Inclusive

**DOI:** 10.1093/geroni/igab046.1979

**Published:** 2021-12-17

**Authors:** Jennifer Drost, Margaret Sanders

## Abstract

As the US population ages, the prevalence of people living with dementia will also increase. It is estimated that by 2050, 13.8 million American’s 65 and older will be diagnosed with dementia, and currently only 40% of those living with dementia receive an official diagnosis. 70% of people living with dementia live in the community. In order to optimize quality of life and extend each person’s ability to remain living in their homes for as long as possible, it is important for communities to educate consumers and providers alike about Alzheimer’s Disease and related dementias, focusing on behaviors and interventions. This education must cross multiple sectors to effectively increase awareness, decrease stigma, and enable participation in community living for people living with dementia and their caregivers. Dementia Friends USA offers a framework for implementation of dementia friendly inclusive community initiatives that spans professions and incorporates patient and caregiver perspectives. The four symposia will 1) lead us through the evidence that supports the Dementia Friends USA approach, 2) demonstrate how this approach can be operationalized in a truly integrated fashion at the community level using HRSA’s Geriatric Workforce Enhancement Program (GWEP), 3) provide step-by-step instructions for implementing Dementia Friends Community sessions, focusing on one sector at a time (in this case the Developmental Disability population), and 4) discuss the individual and community level outcomes of Dementia Friends implementation.

